# Physicochemical property, volatile flavor quality, and microbial community composition of Jinhua fatty ham and lean ham: A comparative study

**DOI:** 10.3389/fmicb.2023.1124770

**Published:** 2023-01-27

**Authors:** Jin Zhang, Ke Zhao, Huanhuan Li, Shuangxi Li, Weimin Xu, Lihong Chen, Jing Xie, Honggang Tang

**Affiliations:** ^1^State Key Laboratory for Managing Biotic and Chemical Threats to the Quality and Safety of Agro-Products, Institute of Food Science, Zhejiang Academy of Agricultural Sciences, Hangzhou, Zhejiang, China; ^2^Xingzhi College, Zhejiang Normal University, Jinhua, Zhejiang, China; ^3^Jinhua Jinnian Ham Co., Ltd., Jinhua, Zhejiang, China; ^4^Zhejiang Institute of Product Quality and Safety Science, Hangzhou, Zhejiang, China

**Keywords:** Jinhua ham, fatty ham, lean ham, volatile flavor compound, bacterial community, fungal community, GC-IMS, high-throughput sequencing

## Abstract

The physicochemical property, volatile flavor compounds, and microbial community structure of Jinhua fatty ham (FH) and lean ham (LH) were investigated and compared by high-throughput sequencing and HS-GC-IMS. Results showed that FH had higher pH and slightly lighter and yellower color than LH. Meanwhile, 33 volatile flavor compounds were identified from FH and LH, among which LH showed higher abundance of total alcohols and acids, but FH had generally richer aldehydes, ketones, esters, heterocyclic, and sulfur-containing compounds. Moreover, FH and LH did not have significant difference in α-diversity of bacterial community, but LH presented a much lower α-diversity of fungal community than FH. Besides, the dominant microorganisms (relative abundance >2%) in FH were *Ruminococcaceae* UCG-005, *Staphylococcus*, *Ruminococcaceae* UCG-014, *Meyerozyma*, and *Aspergillus* at the genus level, while in LH were *Staphylococcus*, *Psychrobacter*, *Halomonas*, *Propionicicella*, *Ruminococcaceae* UCG-005, *Meyerozyma*, *Yamadazyma*, and *Aspergillus*. Furthermore, the analysis of Pearson’s correlation and metabolic network confirmed that the discriminative flavor compounds of FH were mainly β-oxidation and degradation products of fatty acids, while those of LH were mostly derived from the Strecker reaction or microbial metabolism of amino acids. The present study could help understand the potential pathway of characteristic microorganisms affecting flavor formation of fat-deficient dry-cured hams and provide theoretical supports for developing healthier fermented meat products.

## 1. Introduction

Chinese dry-cure ham is a traditional fermented meat product with a long history of over 1,000 years (Li et al., 2022). Jinhua ham, considered one of the most representative Chinese dry-cured hams, is widely prevalent around the world due to its unique flavor and abundant nutrients (Li W. et al., 2021; Wang et al., 2021). Jinhua hams are produced from fresh hind legs of pigs through a long processing procedures, mainly including raw material selection, salting, dry-ripening, and post-ripening, which usually spend several months (Zhou et al., 2021a). The fats, proteins, and glycogens in raw hams can be degraded into large amounts of amino acids, fatty acids, and pyruvates during the dry-ripening, which further produce the characteristic flavor compounds of dry-cured hams through diverse chemical reactions, such as β-oxidation, deamination, Strecker degradation, and Millard reaction (Martínez-Onandi et al., 2017; Bosse et al., 2018; Shi et al., 2019). Hence, fat plays a crucial role in the formation of unique flavor of Jinhua hams. However, high-level fats or polyunsaturated fatty acids in meats are sensitive to oxidative deterioration, which are related with the rancid smell and may have a side effect on consumers’ acceptance (Benet et al., 2015; Zhou et al., 2021b). In addition, the consumption of foods with high-level saturated fats could be associated with an increased risk of cholesterol-related cardiovascular pathologies (Trevisan et al., 1990; Benet et al., 2016). Therefore, the lean ham (LH), manufactured from the pork hind leg removed skin and fat tissues, are more and more popular among consumers, especially populations with health concerns. Nevertheless, the physicochemical property and flavor quality were rarely studied for LH, even though sufficiently reported for normal fatty hams (FH). Moreover, the effect of fat deficiency on the flavor characteristics of dry-cured hams was also not investigated yet.

On the other hand, the aforementioned complex reactions producing unique flavor compounds are dependent on the enzymatic actions of not only endogenous enzymes but also microbial enzymes (Petrova et al., 2015; Wang et al., 2021). Recent studies reported that various microorganisms could promote the formation of characteristic flavor and quality property of dry-cured hams by performing proteolysis, lipolysis, and oxidation activities (Toledano et al., 2019; Chen et al., 2021; Li et al., 2022). Meanwhile, several reports focused on the composition of microbial communities and relative content of volatiles in Jinhua FH (Ge et al., 2017; Wang et al., 2021; Deng et al., 2022). Moreover, the core microorganisms in Jinhua FH, such as *Staphylococcus*, *Lactobacillus*, *Debaryomyces*, and *Apiotrichum*, have been identified positively associated with the flavor compounds derived from the catabolism of amino acids, such as some branched aldehydes and heterocyclic compounds (Zhou et al., 2022). However, there is still a lack of comprehensive knowledge on microbial community of Jinhua LH. Furthermore, the potential metabolic pathway of core bacteria/fungi affecting flavor formation in LH was also not understood.

Therefore, the objective of this study was firstly, to compare the physicochemical property, volatile flavor quality, and microbial community composition of Jinhua FH and LH, and secondly, to explore the correlations and connections between core microbes and differential flavor compounds. The color, pH, water activity, chemical composition, and nitrite residue of both FH and LH were determined. The headspace-gas chromatography-ion mobility spectrometry (HS-GC-IMS) and high-throughput sequencing of 16S rRNA/ITS genes were also utilized to analyze volatile flavor profiles and bacterial/fungal communities, respectively. Furthermore, the analysis of correlation and metabolic network was applied for the exploration of relationship between core microorganisms and volatiles. The information obtained from this study could help understand the potential pathway of characteristic microorganisms influencing flavor formation of dry-cured hams under the condition of fat deficiency.

## 2. Materials and methods

### 2.1. Processing and sampling of Jinhua fatty ham and lean ham

The Jinhua hams used in the present study were prepared and sampled in Jinhua Jinnian Ham Co., Ltd (Zhejiang, China). Six fresh hind legs (14.5 ± 0.5 kg, pH = 5.9 ± 0.2) of domestic pigs (Large White × Landrace) were used to prepare Jinhua FH and LH following the procedures of Zhou et al. (2021a) with some modifications. For Jinhua FH, three whole hind legs were salted for 75 days with 0.014% NaNO_2_ and 10% NaCl per leg, followed by soak cleaning for 1 day and sun-drying for 1 day. Subsequently, hind legs were dehydrated for 7 days in a dehydration plant, sun-dried for another day, and then ripened for 180 days in a dry-ripening room. During the ripening, the ambient temperature progressively increased from 5°C to 35°C, while the relative humidity gradually decreased from 85 to 65%. Afterward, legs were further post-ripened for about 30 days at room temperature (25°C), and the hams were finally obtained when the weight loss reached approximately 40% of the initial weight. For Jinhua LH, the skins and fat tissues were firstly trimmed off from the other three hind legs. The final hams were also obtained following the gradual procedures of salting, soak cleaning, sun-drying, dehydrating, secondary sun-drying, dry-ripening, and post-ripening, which were all performed under the same conditions with Jinhua FH. Furthermore, as described by Chen et al. (2021) with minor modifications, approximately 5 cm × 2 cm × 0.2 cm pieces were cut from the surface of the *biceps femoris* muscle of each ham, which were used for the fungal community determination. Meanwhile, about 20 g interior samples were taken from the central fraction (about 3–4 cm depth) of the *biceps femoris* muscle of each ham, which were used for the evaluation of physicochemical parameters, volatile flavor compounds, and bacterial community. All samples were vacuum-packaged and frozen at-80°C until further analysis.

### 2.2. Measurement of color, pH, and water activity (aw)

The color, pH and water *a*_w_ were measured following the methods of our previous studies (Li H. et al., 2021; Zhang et al., 2022) with minor modifications. The color of samples was directly detected using a colorimeter (NH310, 3NH Technology Co., Ltd., China), where the *L*, a*, and b* values represent lightness, redness/greenness, and yellowness/blueness, respectively. A white standard plate with the *L*, a*, and b* values of 99.46, 0.19, and −1.98, respectively, was used for calibration before color detection. The pH value was determined using a portable pH meter (Testo 205, Testo Instruments Co., Ltd., Shenzhen, China) equipped with a piercing pH probe and a temperature-compensated temperature probe. The *a*_w_ value was determined with an intelligent water activity meter (HD-4, Huake Instrument and Meter Co., Ltd., Wuxi, China).

### 2.3. Detection of chemical composition

The chemical composition of ham samples was detected as described by our previous study (Zhang et al., 2017) with minor modifications. Briefly, the total contents of moisture, protein, fat, and minerals were analyzed by the method of direct drying (GB/T 5009.3–2016), Kjeldahl (ISO 5983-1997), Soxhlet extraction (ISO 1444-1996), and dry-ashing (ISO 5984-2002), respectively.

### 2.4. Estimation of nitrite residue

According to the method of Liu et al. (2019) with minor modifications, the content of residual nitrite was detected with a nitrite assay kit following the manufacturer’s instruction (Nanjing Jiancheng Bioengineering Institute, Nanjing, China). Briefly, ham samples were dispersed into distilled water and filtrated, followed by the reaction with sulphanilamide solution and N-naphthyl-ethylenediamine dihydrochloride. The nitrite content was calculated based on the absorbance of supernatant at 550 nm, which was assayed using an ultraviolet–visible spectrophotometer (UV-1750, Shimadzu, Kyoto, Japan).

### 2.5. Determination of volatile flavor compounds

As described by Liu et al. (2020) with minor modifications, the volatile flavor compounds of ham samples were identified by a headspace-gas chromatography-ion mobility spectrometer (HS-GC-IMS; Flavorspec, G.A.S. Instrument, Germany) equipped with a SE-54 capillary column (15 m × 0.53 mm × 1 μM). Samples were firstly cut into approximately 1 cm × 1 cm × 1 cm cubes and minced by an analytical grinder (Aika Instrument Equipment Co., Ltd., Guangzhou, China). Then 2 g samples were put into a 20 ml headspace sampling vial and incubated at 60°C for 20 min. Afterward, 500 μL headspace was injected into the injector automatically with a heated syringe at 85°C. Subsequently, samples were transferred into the capillary column by high-purity nitrogen (>99.99%) at the following programmed flow rates: initially 2 ml/min for first 2 min, then 10 ml/min for 8 min, next 100 ml/min for 10 min, and eventually 150 ml/min for 5 min. Meanwhile, the temperature of column and drift tube was kept as 60°C and 45°C, respectively, and the flow rate of drift gas (nitrogen gas, >99.99% purity) was maintained as 150 mL/min.

The IMS data were analyzed using the instrumental laboratory analysis view (LAV) software with the Reporter, Gallery Plot, and Dynamic PCA plug-in applications. The volatile flavor compound was identified by comparing the retention index (RI) and drift time (DT) with the NIST library and IMS database retrieval software obtained from G.A.S. The intensities of these compounds were calculated based on the height of selected signal peaks.

### 2.6. Analysis of bacterial and fungal communities

The analysis of microbial communities, including the compositions of both fungi and bacteria, were performed by high-throughput sequencing following the procedures of Chen et al. (2021) and Wang et al. (2021) with some modifications. The total genome DNA of bacteria and fungi was extracted using a Cetyltrimethylammonium Bromide (CTAB) method following the manufacturer’s instructions of the genomic DNA extraction kit. The V3-V4 hypervariable regions of bacterial 16S rRNA genes were amplified with the forward primer 5’-CCTAYGGGRBGCASCAG-3′ and reverse primer 5′-GGACTACNNGGGTATCTAAT-3′. The ITS1-1\u00B0F regions of fungal ITS genes were amplified with the forward primer 5′-CTTGGTCATTTAGAGGAAGTAA-3′ and reverse primer 5′-GCTGCGTTCTTCATCGATGC-3′. All PCR reactions were conducted in 30 μL reactions with 15 μL Phusion® High-Fidelity PCR Master Mix (New England Biolabs), 0.2 μM forward and reverse primers, and approximately 10 ng template DNA. PCR products were then mixed in equidensity ratios and purified through the Axy Prep DNA Gel Extraction Kit (AXYGEN; for bacteria) or Qiagen Gel Extraction Kit (Qiagen, Germany; for fungi).

The data sequencing was carried out according to the procedures of Wang et al. (2021) and Li et al. (2022) with some modifications. Sequencing libraries were generated using the NEB Next® Ultra™ DNA Library Prep Kit for Illumina (NEB, United States; for bacteria) or TruSeq® DNA PCR-Free Sample Preparation Kit (Illumina, United States; for fungi) following the manufacturer’s recommendations and index codes were added. The library quality was evaluated on the Qubit@ 2.0 Fluorometer (Thermo Fisher Scientific) and Agilent Bioanalyzer 2,100 system. Then the library was sequenced on the Illumina Miseq/HiSeq2500 (for bacteria) or NovaSeq (for fungi) platform. Afterward, the QIIME (V1.9.1) quality controlled process was performed to filter raw reads to obtain high-quality clean reads. The reads were compared with the reference database (Silva database) using the UCHIME algorithm to detect chimera sequences, and the chimera sequences were removed to acquire the effective clean reads. The sequence analysis was performed using the UPARSE algorithms. Sequences with ≥97% similarity were assigned to the same operational taxonomic units (OTUs), and representative sequences for each OTU were screened for further annotation. The significance of differences in microbial communities was statistically analyzed based on the relative abundance of bacteria or fungi at various levels, mainly including phylum, genus, and species. The α-diversity was presented *via* the Shannon index, Simpson index, ACE index, Chao 1 index, and observed species, while the β-diversity was assayed by the principal coordinate analysis (PCoA).

### 2.7. Statistical analysis

All experiments were performed in triplicate with the results shown as average ± standard deviation. Tables were made by the Microsoft Excel 2016 software, while figures were drawn with the Origin V2021 (Origin-Lab, Northampton, United States) and Microsoft PowerPoint 2016. Analysis of variance (ANOVA) was performed using the SAS V8 software (SAS Institute Inc., Carry, United States), while analysis of Pearson’s correlation and hierarchical cluster (HCA) was conducted by the plug-in applications in Origin V2021. Differences among mean values were established with the Duncan multiple range test. The significant difference was confirmed when *p* < 0.05.

## 3. Results and discussion

### 3.1. Differences In physicochemical properties

The physicochemical property of dry-cure ham was closely associated with the flavor quality and microbial diversity (Domínguez et al., 2022). The physicochemical parameters of internal samples from Jinhua FH and LH, including color, water activity (*a*_w_), pH, chemical composition, and nitrite residue, are shown in Table 1. It is clear that there is no significant distinction between the a* values (redness) of FH and LH (*p* > 0.05). However, FH showed slightly higher *L* and b* values than LH (*p* < 0.05), suggesting that the color of FH was slightly lighter and yellower than that of FH. This result may be attributed to their difference in the redox degree of proteins, especially the myoglobin and hemoglobin (Parolari et al., 2016). Specifically, myoglobin and hemoglobin are the main pigments responsible for the color of dry-cured hams (Parolari et al., 2016; Zhou et al., 2021b), and the lack of protection by fat and skin tissues might allow more myoglobins/hemoglobins in LH to be oxidized during dry-ripening, possibly resulting in the darker color of LH.

**Table 1:** Physicochemical parameters of internal samples from Jinhua FH and LH.

Physicochemical indexes	FH	LH
*L*	29.44 ± 0.89^a^	25.33 ± 0.36^b^
a*	12.38 ± 0.11^a^	12.11 ± 0.27^a^
b*	5.27 ± 0.83^a^	3.43 ± 0.85^b^
*a*_w_	0.71 ± 0.09^a^	0.73 ± 0.01^a^
pH	6.08 ± 0.03^a^	5.75 ± 0.04^b^
Moisture (%)	31.90 ± 6.47^a^	31.37 ± 3.88^a^
Protein (%)	46.53 ± 3.75^a^	44.10 ± 1.87^a^
Mineral (%)	8.43 ± 1.05^a^	8.67 ± 1.06^a^
Fat (%)	4.60 ± 0.80^a^	3.23 ± 0.91^a^
Nitrite (mg/kg)	1.13 ± 0.21^a^	1.57 ± 0.12^a^

Different lowercases within the same row denote significant differences between Jinhua FH and LH (*p* < 0.05). FH, fatty ham; LH, lean ham.

**Figure 1:**
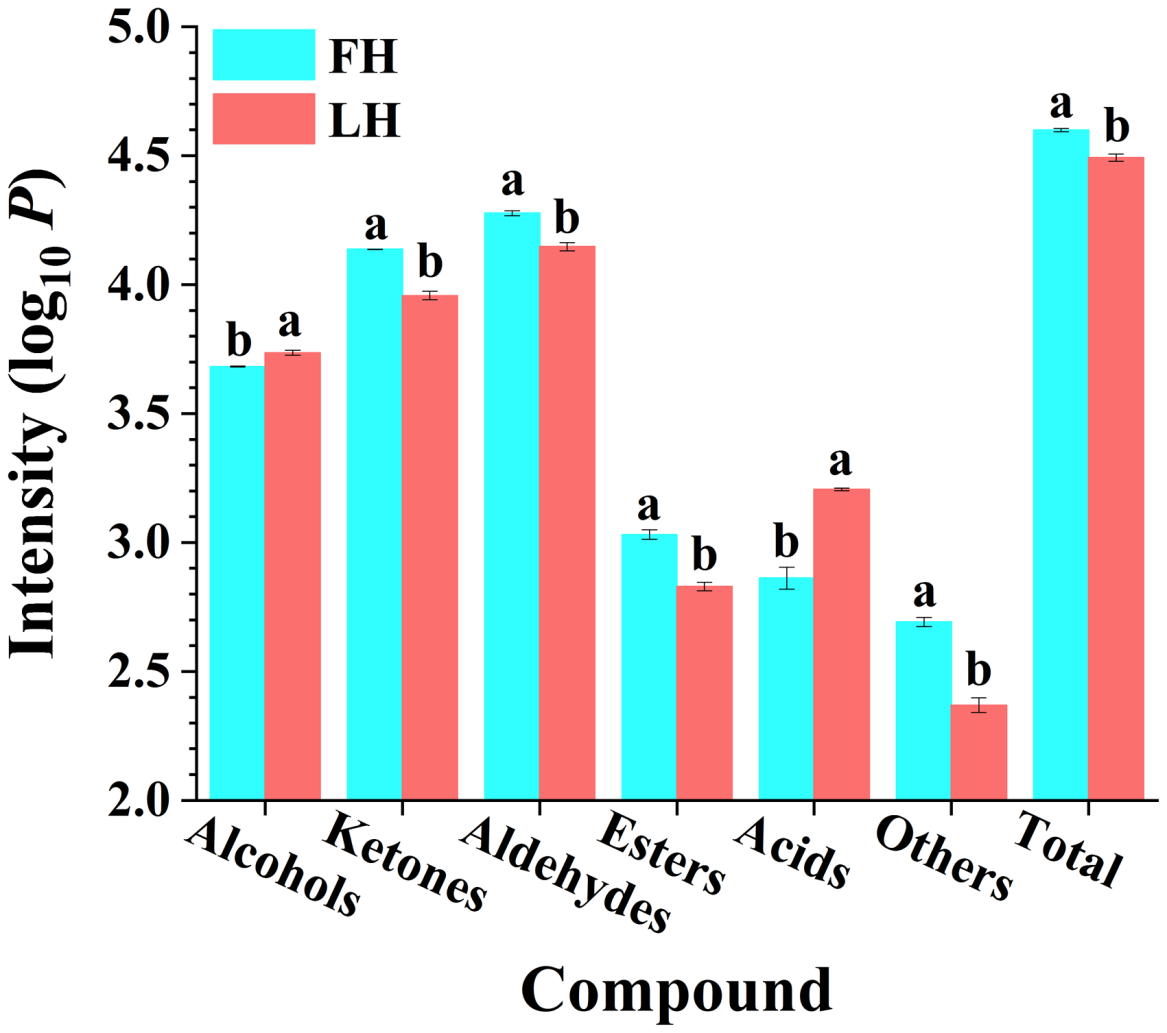
Comparison of volatile flavor compound intensities of Jinhua FH and LH in different chemical families. Different lowercases above the error bar denote significant differences between Jinhua FH and LH (*p* < 0.05). FH, fatty ham; LH, lean ham. Log_10_ *P* is the logarithmic value of total intensity for compounds of each chemical family in HS-GC-IMS spectra.

Besides, the *a*_w_ values of FH and LH were not obviously different (*p* > 0.05) and both far under 0.85, which was a low *a*_w_ that protects hams from most harmful microbes and meanwhile allows the growth of salt-consuming and flavor-generating bacteria/fungi (Chen et al., 2021). However, LH exhibited a remarkable lower pH than FH (*p* < 0.05). Bosse et al. (2018) reported that a pH of 6.0–6.2 would cause a higher microbial risk and reduced water diffusion ability in hams, while hams with pH of 5.6–6.0 have more desirable saltiness, color, and texture. The pH values of FH and LH were 6.08 ± 0.03 and 5.75 ± 0.04, respectively, suggesting that LH might has higher sensory quality and edible safety than FH. The distinct pH values of FH and LH was probably attributed to their distinction in the intensity of acids generated during ripening (Table 2; Figure 1).

**Table 2:** Intensities of 33 identified volatile flavor compounds from Jinhua FH and LH by HS-GC-IMS.

Volatile flavor compound	RI	RT (s)	DT (ms)	Intensity (V)		Odor description
				FH	LH	
Alcohols (7)						
1-Octen-3-ol (M)	984.1	564.06	1.159	503.76 ± 6.15^a^	274.88 ± 13.82^b^	Mushroom, earthy, green, oily, fungal, and raw chicken
1-Pentanol (M)	764.0	250.95	1.252	493.27 ± 12.62^a^	412.24 ± 11.95^b^	Fusel oil, sweet, and balsam
1-Pentanol (D)	761.8	248.94	1.504	199.36 ± 6.12^a^	105.39 ± 3.98^b^	
Ethanol (M)	460.0	102.79	1.047	1002.96 ± 11.62^a^	417.87 ± 6.27^b^	Strong alcoholic, ethereal, and medical
2-Propanol (M)	492.9	112.30	1.093	797.08 ± 20.78^b^	2096.11 ± 73.43^a^	Alcohol, musty, and woody
1-Hexanol (M)	868.8	364.51	1.639	71.06 ± 0.37^a^	30.14 ± 4.30^b^	Ethereal, fusel oil, fruity, alcoholic, and sweet green
3-Hexen-1-ol (M)	854.8	346.98	1.233	45.35 ± 3.40^b^	179.71 ± 2.83^a^	Fresh, green, cut grass, foliage vegetable, herbal, and oily
3-Hexen-1-ol (D)	855.1	347.34	1.515	27.70 ± 6.54^b^	95.37 ± 12.43^a^	
3-Methyl-1-butanol (M)	729.6	221.15	1.246	679.72 ± 7.45^b^	1049.00 ± 13.76^a^	Fusel oil, alcoholic, whiskey, fruity, and banana
3-Methyl-1-butanol (D)	732.4	223.45	1.488	990.89 ± 14.04^a^	787.58 ± 7.39^b^	
Ketones (8)						
3-Octanone (M)	990.4	577.91	1.303	159.31 ± 13.96^a^	72.22 ± 0.52^b^	Fresh, herbal, lavender, sweet, and mushroom
6-Methyl-5-hepten-2-one (M)	990.9	578.83	1.178	65.32 ± 2.46^a^	43.86 ± 3.84^b^	Citrus, green, musty, lemongrass, and apple
2-Octanone (M)	995.4	589.04	1.334	115.78 ± 6.85^a^	67.28 ± 2.40^b^	Earthy, weedy, natural woody, and herbal
2-Heptanone (M)	890.9	394.27	1.261	660.05 ± 11.70^a^	320.17 ± 15.8^b^	Fruity, spicy, sweet, herbal, coconut, and woody
2-Heptanone (D)	890.3	393.34	1.636	460.71 ± 20.68^a^	155.21 ± 1.46^b^	
3-Hydroxy-2-butanone (M)	706.1	202.82	1.051	511.53 ± 11.82^a^	320.74 ± 2.83^b^	Sweet, buttery, creamy, dairy, milky, and fatty
3-Hydroxy-2-butanone (D)	706.6	203.21	1.331	347.91 ± 6.76^a^	172.67 ± 2.35^b^	
2-Butanone (M)	582.2	142.82	1.061	852.91 ± 27.62^a^	712.45 ± 17.96^b^	Acetone-like, ethereal, fruity, and camphor
2-Butanone (D)	582.2	142.82	1.249	5370.31 ± 127.24^a^	2892.93 ± 88.18^b^	
Acetone (M)	489.2	111.19	1.122	4875.18 ± 109.71^a^	3946.4 ± 204.50^b^	Solvent, ethereal, apple, and pear
2-Pentanone (M)	684.0	187.89	1.376	299.59 ± 26.72^a^	370.21 ± 49.84^a^	Sweet, fruity, ethereal, wine, banana, and woody
Aldehydes (10)						
n-Nonanal (M)	1103.1	790.53	1.473	458.85 ± 18.37^a^	394.49 ± 28.14^b^	Waxy, aldehydic, rose, fresh, orris, orange, peel, fatty, and peely
Phenylacetaldehyde (M)	1046.9	678.37	1.253	838.99 ± 87.35^b^	1025.35 ± 21.09^a^	Green, sweet, floral, hyacinth, clover, honey, and cocoa
Benzaldehyde (M)	957.3	508.64	1.152	701.51 ± 14.23^a^	358.10 ± 3.70^b^	Strong, sharp, sweet, bitter, almond, and cherry
Benzaldehyde (D)	957.6	509.33	1.472	287.89 ± 18.00^a^	111.62 ± 11.07^b^	
Heptanal (M)	899.5	407.29	1.332	735.02 ± 10.05^a^	676.13 ± 63.83^a^	Fresh, aldehydic, fatty, green, herbal, wine-lee, and ozone
Heptanal (D)	898.7	406.05	1.700	339.87 ± 24.74^a^	364.63 ± 95.29^a^	
Hexanal (M)	789.8	275.65	1.564	3640.15 ± 347.86^a^	3153.10 ± 219.67^a^	Fresh, green, fatty, aldehydic, grass, leafy, fruity, and sweaty
Pentanal (M)	696.8	196.03	1.181	526.97 ± 25.28^a^	426.90 ± 12.33^b^	Fermented, bready, fruity, nutty, and berry
Pentanal (D)	693.4	193.58	1.422	337.35 ± 68.60^a^	457.12 ± 83.29^a^	
2-Methylbutanal (M)	661.6	176.87	1.403	3545.00 ± 9.58^a^	2102.76 ± 28.43^b^	Musty, cocoa, phenolic, coffee, nutty, malty, fermented, fatty, and alcoholic
3-Methylbutanal (M)	643.0	168.25	1.411	5331.26 ± 80.80^a^	3386.63 ± 13.16^b^	Ethereal, aldehydic, chocolate, peach, and fatty
Octanal (M)	1009.5	612.88	1.406	487.59 ± 18.22^a^	510.4 ± 63.07^a^	Aldehydic, waxy, citrus, orange, peel, green, herbal, fresh, and fatty
Octanal (D)	1009.0	612.04	1.831	61.05 ± 5.04^a^	69.99 ± 13.53^a^	
2-Methylpropanal (M)	550.3	131.08	1.107	1110.38 ± 27.14^a^	614.7 ± 4.31^b^	Fresh, aldehydic, floral, and green
2-Methylpropanal (D)	559.6	134.41	1.282	513.10 ± 60.84^a^	384.57 ± 8.87^b^	
Esters (4)						
γ-Butyrolactone (M)	915.5	433.14	1.086	172.75 ± 35.18^a^	172.77 ± 13.88^a^	Creamy, oily, fatty, and caramel
Ethyl acetate (M)	614.6	155.86	1.103	287.71 ± 9.74^a^	160.31 ± 7.09^b^	Ethereal, fruity, sweet, weedy, and green
Ethyl acetate (D)	618.8	157.63	1.338	477.6 ± 19.91^a^	213.95 ± 21.01^b^	
Butyl acetate (M)	808.2	294.23	1.237	93.36 ± 3.70^a^	65.08 ± 3.88^b^	Ethereal, solvent, fruity, and banana
Butyl acetate (D)	806.9	292.80	1.622	18.75 ± 8.54^a^	20.13 ± 3.71^a^	
Butyl propanoate (M)	908.9	422.32	1.281	24.65 ± 9.19^b^	43.59 ± 1.69^a^	Earthy, sweet, weak, and rose
Acids (2)						
Isovaleric acid (M)	837.4	326.20	1.208	450.36 ± 63.62^b^	643.21 ± 22.42^a^	Sour, stinky, feet, sweaty, cheese, and tropical
Isovaleric acid (D)	837.4	326.20	1.491	52.44 ± 8.41^b^	88.37 ± 3.49^a^	
2-Methylpropanoic acid (M)	750.8	239.03	1.162	174.86 ± 5.30^b^	477.95 ± 5.84^a^	Acidic, sour, cheese, dairy, buttery, and rancid
2-Methylpropanoic acid (D)	752.8	240.79	1.377	51.77 ± 9.65^b^	397.47 ± 9.53^a^	
Heterocyclic (1)						
2-Pentylfuran (M)	993.0	583.67	1.255	255.32 ± 17.21^a^	162.83 ± 13.59^b^	Fruity, green, earthy, beany, vegetable, and metallic
Sulfur-containing (1)						
Dimethyl disulfide (M)	746.1	234.96	0.970	237.54 ± 3.56^a^	71.35 ± 2.33^b^	Sulfurous, vegetable, cabbage, and onion

Different lowercases within the same row denote significant differences between Jinhua FH and LH (*p* < 0.05). FH, fatty ham; LH, lean ham; RI, retention index; RT, retention time; DT, drift time; M, monomer; D, dimer. The odor description of volatile flavor compounds was from the good scents company information system (www.thegoodscentscompany.com).

Furthermore, these two hams also exhibited no significant difference in chemical compositions (*p* > 0.05). The moisture content of both FH and LH (around 31.5%) was similar with that of Mianning hams reported by Chen et al. (2021), which is low enough to prevent spoilage and improve quality of hams. This result was also consistent with the *a*_w_ data as mentioned above, since the water content is usually considered positively correlated with the water activity. Meanwhile, the minerals in hams can be mainly salts (Domínguez et al., 2022), hence FH and LH showed similar mineral contents (about 8.50%; *p* > 0.05) because of the same adding amount of NaCl during salting. Additionally, the nitrite residues were 1.13 ± 0.21 and 1.57 ± 0.12 for FH and LH, respectively, without marked distinction (*p* > 0.05) and far under the secure nitrite residue limits (30 mg/kg) for meat products in China (Chen et al., 2021). On the other hand, the pigments contributing to the rose-red color of dry-cured hams are mainly nitromyoglobins and nitrohemoglobins, which are the product of reactions between nitrites/nitrates and myoglobins/hemoglobins (Parolari et al., 2016). Therefore, the similar nitrite residue of FH and LH suggested a same nitrite consuming amount for the production of red pigments, which can be the main reason for their similar redness (a* value) as mentioned above. Overall, the data shown in Table 1 indicate that LH had an obviously lower lightness, yellowness, and pH than FH, probably resulting from their discrimination in oxidation and fermentation products.

### 3.2. Comparison of volatile flavor compounds

**Figure 2:**
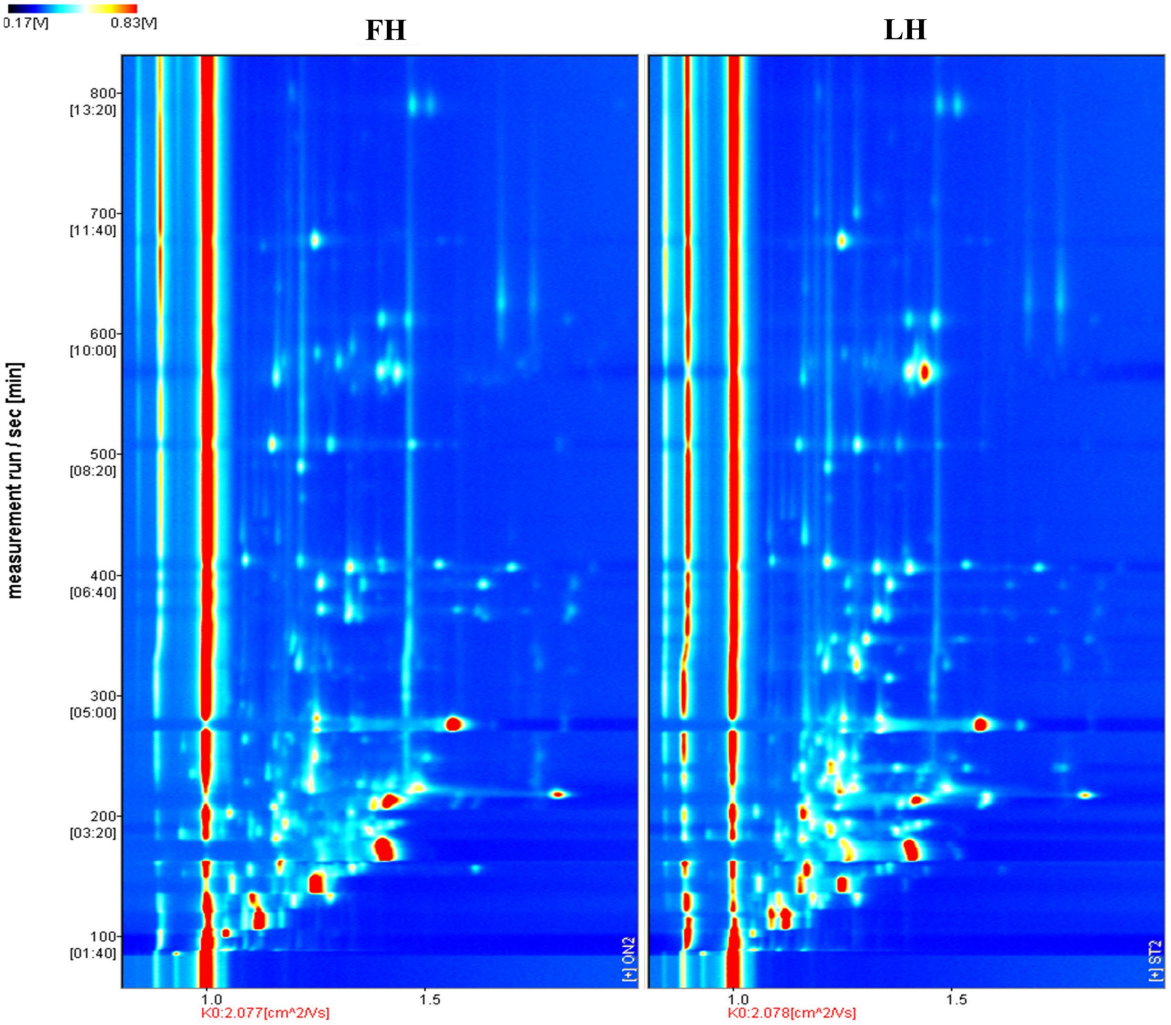
Comparison of volatile flavor profiles of Jinhua FH and LH with two-dimension HS-GC-IMS spectra. FH, fatty ham; LH, lean ham.

The volatile flavor profiles of internal samples from Jinhua FH and LH were analyzed by HS-GC-IMS and results are illustrated as a two-dimension spectra plot (Figure 2). The *y*-axis and *x*-axis in Figure 2 showed the retention time (RT) of GC and the relative drift time (DT) of ions, respectively (Liu et al., 2020). Meanwhile, the red line representing *x* = 1.0 was the reactive ion peak (RIP). Besides, each data point corresponded to a volatile flavor compound, and its color indicated the intensity of volatile (Li et al., 2019). Specifically, the blue and white colors represented a low intensity of volatiles, whereas the yellow and red colors showed a high intensity of compounds. It is exhibited that the two hams showed similar number of ion peaks within the DT range of 1.0–1.7 ms, suggesting that the numbers of main identified volatiles were not obviously different in FH and LH. However, the data points (ion peaks) of FH mostly showed a redder color or bigger size than those of LH, indicating that FH had an overall higher intensity of volatile flavor than LH.

**Figure 3:**
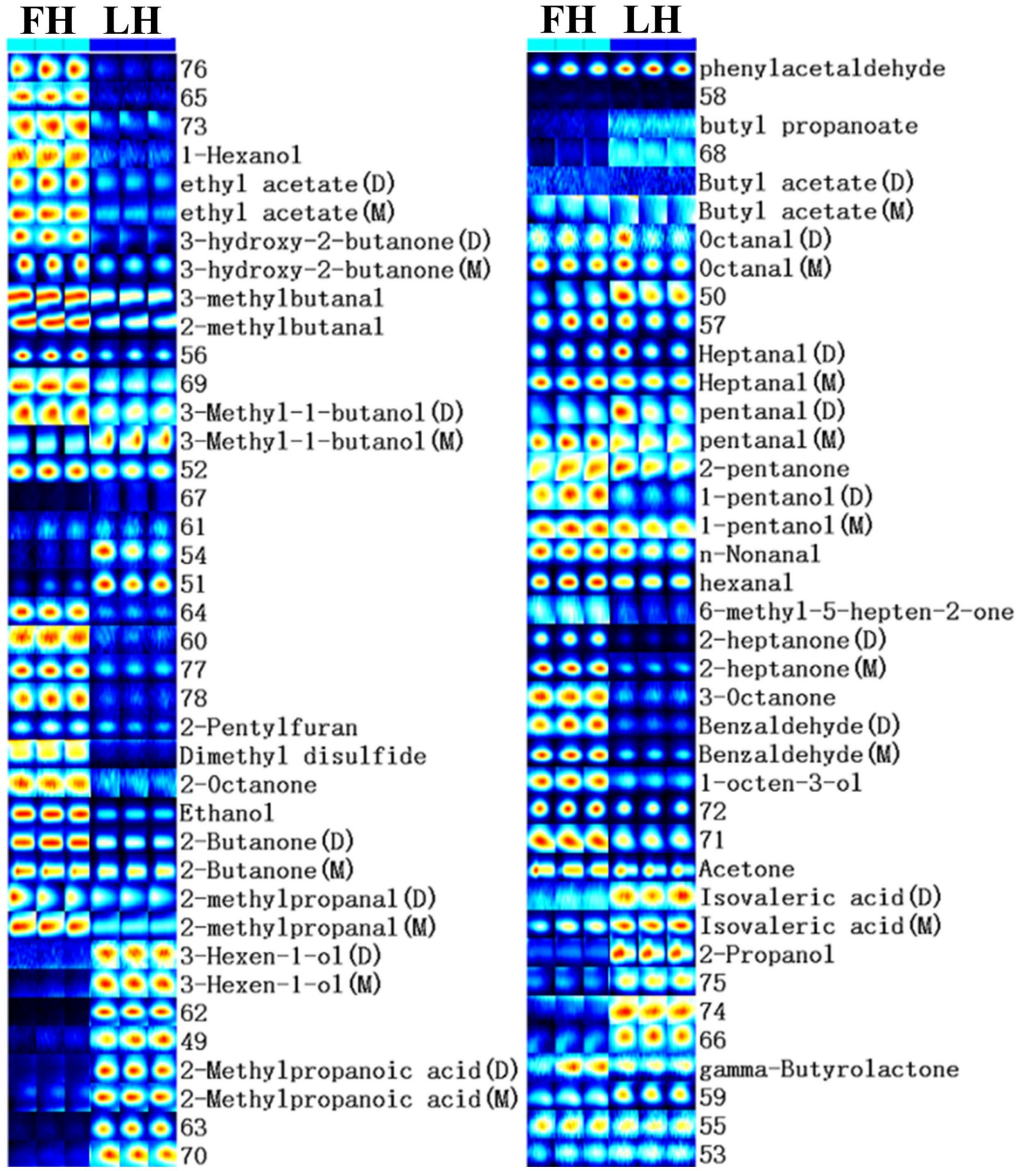
Gallery plot of characteristic flavor finger-print of Jinhua FH and LH determined by HS-GC-IMS. FH, fatty ham; LH, lean ham.

Furthermore, to compare the finger-print of characteristic volatiles in FH and LH, the Gallery plot plug-in was utilized with results presented in Figure 3. Each row in Figure 3 represented a single volatile flavor compound, which could correspond to a single signal (monomer) or a pot (dimer) in the IMS spectra plot (Figure 2) depending on its concentration (Arroyo-Manzanares et al., 2018). As shown in Figure 3, a total of 33 volatile compounds were identified, which can be classified into 7 chemical families. In details, there were 7 alcohols, 8 ketones, 10 aldehydes, 4 esters, 2 acids, 1 heterocyclic compound, and 1 sulfur-containing compound. These findings were in accordance with the reports of Liu et al. (2020), Wang et al. (2021), and Li et al. (2022) on the volatile profiles of Jinhua hams.

The profiles of individual volatile flavor compounds were further analyzed with data shown in Table 2. Aldehydes are known as the major contributors to the unique flavor of fermented meats due to their high concentrations and low aroma thresholds (Liu et al., 2020; Wang et al., 2021). As exhibited in Table 2, hexanal, 2-methylbutanal, and 3-methylbutanal were the richest aldehydes in both FH and LH, followed by 2-methylpropanal (for FH) or phenylacetaldehyde (for LH), in accordance with the findings of Wang et al. (2021) on the volatiles of Jinhua hams. Hexanal is regarded as the major lipid oxidation product in dry-cured hams, and a low-content hexanal generally provides a pleasant grassy, fruity, and green odor (Benet et al., 2015; Domínguez et al., 2022). 2-methylbutanal, 3-methylbutanal, and 2-methylpropanal are all branched aldehydes, deriving from oxidative deamination and decarboxylation of valine, leucine, and isoleucine through Strecker degradation (Narváez-Rivas et al., 2014). Meanwhile, they are also medium-chain (C4-C9) aliphatic aldehydes, which were considered responsible for grassy, fatty, and/or nutty flavors of meat products (Liu et al., 2020; Wang et al., 2021). Furthermore, the abundances of most identified aldehydes were significantly lower (*p* < 0.05) or similar (*p* > 0.05) in LH compared with those in FH. Noteworthily, only the phenylacetaldehyde showed a relatively higher richness in LH (*p* < 0.05). Phenylacetaldehyde is originated from the Strecker degradation of phenylalanine and might contribute to the spicy sensation of hams (García-González et al., 2008).

Ketones are another important flavor component in dry-cured meat products, and the high-intensity ketones are usually associated with the creamy, fruity, cooked, and spicy flavor characteristics (Zhu et al., 2018). The most abundant ketones in the two hams were 2-butanone and acetone (*p* < 0.05; Table 2). Acetone could be mainly transferred from acetyl-CoA, one of the major degradation products of glycogens in the muscle, and imparts a buttery taste and fruity aroma to meat products (Shi et al., 2019; Li W. et al., 2021). 2-butanone and other methyl ketones are produced *via* the decarboxylation of β-keto acids or the β-oxidation of saturated fatty acids, and act as precursors in the formation of fatty flavor during the ripening of meats (Pham et al., 2008; Shi et al., 2019). Besides, FH had significantly higher intensities in almost all identified ketones than LH (*p* < 0.05) excluding 2-pentanone. The 2-pentanone did not show noteworthy distinction between the two hams (*p* > 0.05; Table 2).

Alcohols are considered contributing less to the aroma of hams because of their relatively higher odor thresholds than aldehydes and ketones, but they can also impart to an herbal, woody, and oily flavor at high concentrations (Liu et al., 2020). The linear-chain aliphatic alcohols are the oxidation products of lipids, whereas the branched alcohols are mostly reported as the microbial degradation products of corresponding branched aldehydes (Shi et al., 2019). It is clear in Table 2 that the most abundant alcohol was obviously distinct for the two hams, which was 2-propanol in LH but ethanol in FH. Meanwhile, most identified linear-chain alcohols showed a higher richness in FH (*p* < 0.05), while 2-propanol, 3-methyl-1-butanol (monomer), and 3-hexen-1-ol presented higher abundances in LH (*p* < 0.05).

Two short-chain fatty acids (SCFAs; C1-C6), isovaleric acid and 2-methylpropanoic acid, were also identified in both FH and LH, which probably originated from the deamination of amino acids or the secondary metabolism by microbes (Liu et al., 2020; Li et al., 2022). Interestingly, both the two identified acids were more abundant in LH (*p* < 0.05; Table 2), which can be a key reason for its relatively lower pH (*p* < 0.05; Table 1). These acids could contribute to a sour taste and cheese flavor, and neutralize some deleterious alkaline compounds in fermented meats such as amine and pyrazine (García-González et al., 2008; Liu et al., 2020). Esters were found with relatively high aroma thresholds, generated *via* the esterification between carboxylic acids and alcohols, and partially provided the sweet, fruit, and/or fatty flavors in meat products (Carrapiso et al., 2015). The predominant esters were ethyl acetate and γ-butyrolactone in both FH and LH. The intensity of γ-butyrolactone was similar in FH and LH (*p* > 0.05), but the richness of ethyl acetate was relatively higher in FH than that in LH (*p* < 0.05).

Moreover, only one heterocyclic compound, 2-pentylfuran, was found in the two hams, which was also determined as the most abundant furans in Chinese bone-less hams by Li et al. (2022). 2-pentylfuran is regarded as an odor-active compound with a green and fruity flavor in dry-cured hams (García-González et al., 2008). In addition, dimethyl disulfide was the only identified sulfur-containing volatile in the two hams, which was in line with the report of Liu et al. (2020) on Jinhua hams. This chemical family is generally products of sulfur-containing amino acid catabolism or microbial metabolism, and usually provides an unpleasant flavor with low odor threshold (Pérez-Santaescolástica et al., 2018). However, dimethyl disulfide has a vegetable aroma and important contribution to the characteristic flavors of cured meats (Liu et al., 2020). Besides, both 2-pentylfuran and dimethyl disulfide showed higher concentrations in FH than those in LH (*p* < 0.05).

The total abundance of volatiles belonged to each chemical family was further summarized with results illustrated in Figure 1. It is shown that FH had significantly higher total intensity of ion peaks for volatiles than LH (*p* < 0.05). On one hand, aldehydes were the most abundant volatiles among all chemical families for both FH and LH, followed by ketones and alcohols, which were consistent with the findings of Liu et al. (2020) and Li W. et al. (2021) on volatiles from various Chinese dry-cured hams. On the other hand, LH showed remarkably higher total intensities of acids and alcohols (*p* < 0.05), whereas other types of volatiles were obviously less abundant for LH (*p* < 0.05).

### 3.3. Analysis of bacterial community structure

The high-throughput sequencing of 16S rRNA genes was performed to investigate the bacterial community structures of Jinhua FH and LH. The α-diversity of bacteria from FH and LH, including Shannon, Simpson, ACE, and Chao 1 indexes, are shown in Table 3. Shannon and Simpson indexes represent the community diversity, and the ACE and Chao 1 indexes are associated with the community richness (Mu et al., 2020). It is clear that the two hams did not have significantly different α-diversities in terms of all indexes (*p* > 0.05). Besides, the numbers of observed bacterial species in FH and LH were 1,058 ± 307 and 1,262 ± 71, respectively, without marked difference (*p* > 0.05). These data indicate that FH and LH had an overall similar diversity and richness in bacterial communities.

**Table 3:** **α**-diversity indexes of bacterial communities in Jinhua FH and LH.

α-diversity indexes	FH	LH
Shannon	5.24 ± 0.83^a^	5.23 ± 1.06^a^
Simpson	0.88 ± 0.07^a^	0.83 ± 0.11^a^
ACE	1314.42 ± 269.91^a^	1316.98 ± 62.12^a^
Chao1	1280.03 ± 274.09^a^	1344.48 ± 85.11^a^
Goods coverage	1.00 ± 0.00^a^	1.00 ± 0.00^a^
Observed species	1,058 ± 307^a^	1,262 ± 71^a^

Different lowercases within the same row denote significant differences in bacterial communities between Jinhua FH and LH (*p* < 0.05). FH, fatty ham; LH, lean ham.

**Figure 4:**
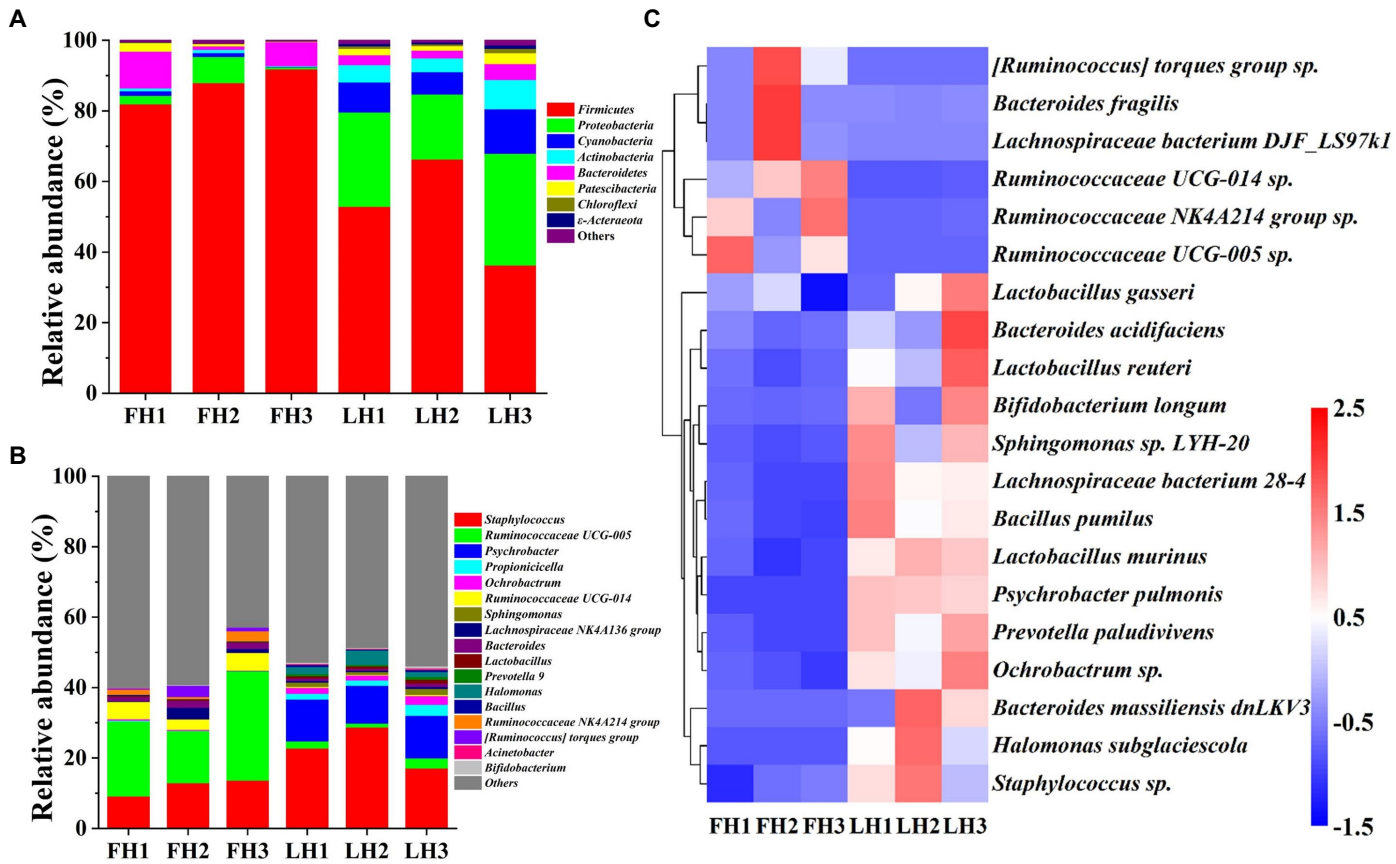
Bacterial community structure of Jinhua FH and LH at the phylum, genus, and species levels. (**A)** Relative abundance at the phylum level. (**B)** Relative abundance at the genus level. (**C)** Heatmap of HCA for the relative abundance of main species (top 20). The color gradation in panel **C** represents the Z-scores of corresponding relative abundances. FH, fatty ham; LH, lean ham.

Figure 4 compares the bacterial community structure of two hams at various levels, including phylum, genus, and species. As illustrated in Figure 4A, *Firmicutes* was the most abundant bacteria at the phylum level in FH with a relative abundance of 87.23% (*p* < 0.05), followed by *Bacteroidetes* (6.06%) and *Proteobacteria* (3.46%; *p* < 0.05). This result was in consistence with the reports of Wang et al. (2021) and Li et al. (2022) on the bacterial composition of Jinhua hams. However, the dominant bacterial phyla (>5%) in LH showed a greater variety, which included *Proteobacteria* (41.39%), *Firmicutes* (20.33%), *Cyanobacteria* (16.26%), *Actinobacteria* (8.92%), and *Bacteroidetes* (5.57%; *p* < 0.05). Compared with the bacteria in FH, the richness of *Firmicutes* was sharply decreased in LH (*p* < 0.05), but *Proteobacteria*, *Cyanobacteria*, *Actinobacteria*, *Chloroflexi*, and *ε-acteraeota* exhibited a significant elevation in relative abundance (*p* < 0.05).

At the genus level (Figure 4B), *Ruminococcaceae* UCG-005 (22.39%), *Staphylococcus* (11.91%), and *Ruminococcaceae* UCG-014 (4.27%) were the dominant bacteria (>2%) in FH (*p* < 0.05), while *Staphylococcus* (22.88%) and *Psychrobacter* (11.58%) were the richest bacterial genera in LH (*p* < 0.05), followed by *Halomonas* (2.50%), *Propionicicella* (2.07%), and *Ruminococcaceae* UCG-005 (2.02%; *p* < 0.05). Wang et al. (2021) reported that *Staphylococcus*, *Psychrobacter*, *Halomonas*, and unidentified *Ruminococcaceae* were among dominant bacterial genera of Jinhua hams ripened for different months, which were corresponded to our above findings. Furthermore, among the main bacterial genera with a relative abundance >1%, FH had richer *Ruminococcaceae* UCG-005, *Ruminococcaceae* UCG-014, *Bacteroides*, *Ruminococcaceae* NK4A214 group, and [*Ruminococcus*] torques group (*p* < 0.05), whereas LH showed higher richness of *Staphylococcus*, *Psychrobacter*, *Propionicicella*, *Ochrobactrum*, *Sphingomonas*, *Lactobacillus*, and *Halomonas* (*p* < 0.05).

At the species level (Figure 4C), the dominant bacteria (>1%) were *Staphylococcus* sp., *Ruminococcaceae* UCG-005 *sp.*, and *Ruminococcaceae* NK4A214 group sp. for FH (*p* < 0.05), whereas *Staphylococcus sp.*, *Ochrobactrum sp.*, *Psychrobacter pulmonis*, and *Halomonas subglaciescola* for LH (*p* < 0.05). On the other hand, among the core bacterial species with a richness >0.5%, the richness of *Ruminococcaceae* UCG-005 sp. and *Ruminococcaceae* NK4A214 group *sp.* were more abundant in FH (*p* < 0.05), while *Staphylococcus sp.*, *Ochrobactrum sp.*, *Psychrobacter pulmonis*, *Prevotella paludivivens*, and *Halomonas subglaciescola* presented a relatively higher richness in LH (*p* < 0.05).

### 3.4. Analysis of fungal community structure

The high-throughput sequencing of ITS genes was applied to analyze the fungal community structures of Jinhua FH and LH. The α-diversity of fungi from the two hams are exhibited in Table 4. Generally, FH presented much higher Shannon, ACE, and Chao 1 indexes than LH (*p* < 0.05). Moreover, the number of fungal species observed from FH was 697 ± 147, approximately 2.8 times of that observed from LH (246 ± 5; *p* < 0.05). These results reveal that both diversity and richness of fungal community in LH was dramatically reduced in comparison to FH.

**Table 4:** **α**-diversity indexes of fungal communities in Jinhua FH and LH.

α-diversity indexes	FH	LH
Shannon	3.58 ± 1.15^a^	1.91 ± 0.03^b^
Simpson	0.66 ± 0.17^a^	0.56 ± 0.02^a^
ACE	750.46 ± 161.03^a^	277.53 ± 13.40^b^
Chao1	742.63 ± 160.02^a^	270.22 ± 13.81^b^
Goods coverage	1.00 ± 0.00^a^	1.00 ± 0.00^a^
Observed species	697 ± 147^a^	246 ± 5^b^

Different lowercases within the same row denote significant differences in fungal communities between Jinhua FH and LH (*p* < 0.05). FH, fatty ham; LH, lean ham.

**Figure 5:**
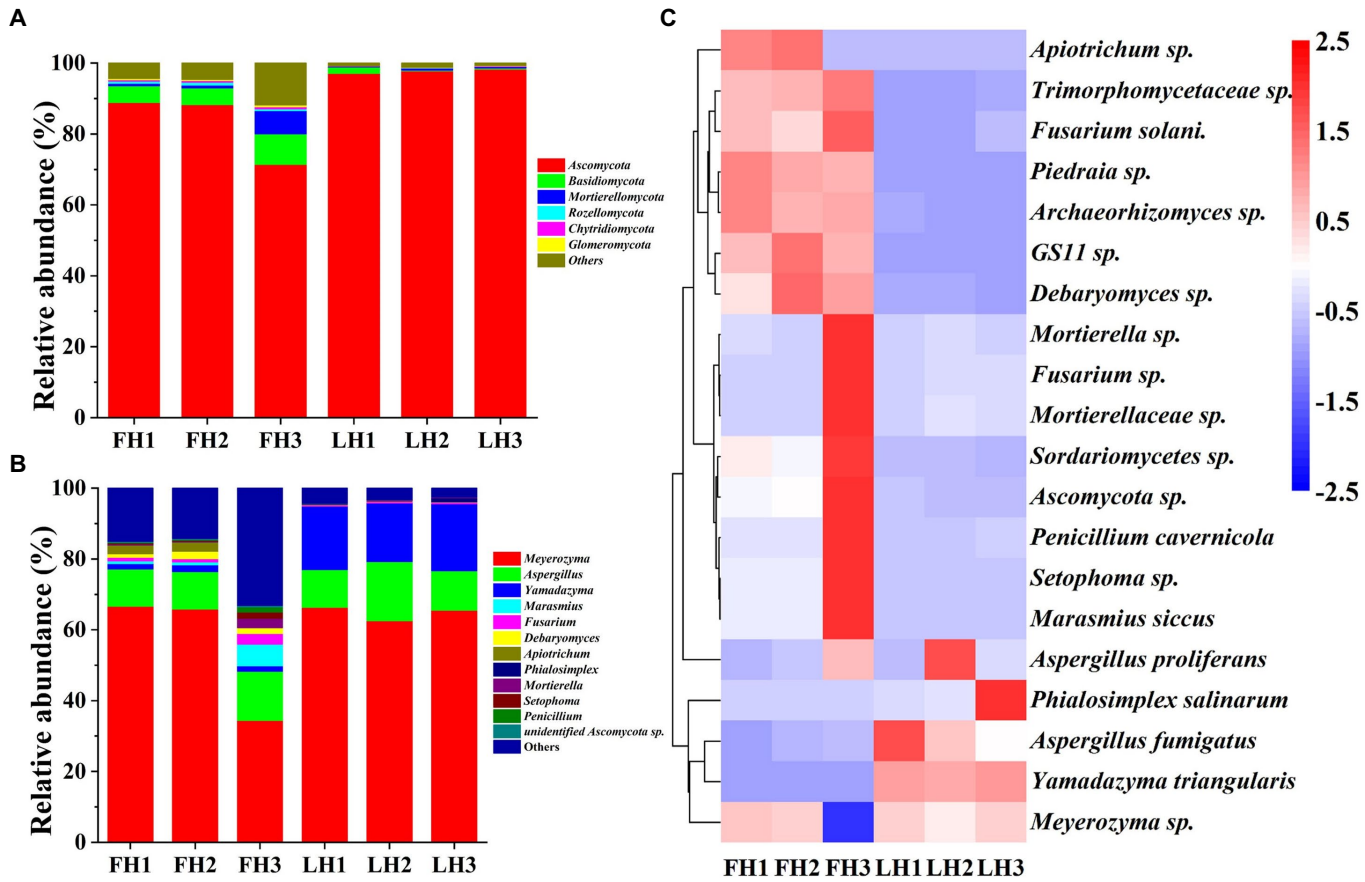
Fungal community structure of Jinhua FH and LH at the phylum, genus, and species levels. **(A)** Relative abundance at the phylum level. **(B)** Relative abundance at the genus level. **(C)** Heatmap of HCA for the relative abundance of main species (top 20). The color gradation in panel **C** represents the Z-scores of relative abundances. FH, fatty ham; LH, lean ham.

Figure 5 illustrates the fungal community structure of FH and LH at the phylum, genus, and species level. As illustrated in Figure 5A, *Ascomycota* was the richest fungus phylum in FH and accounted for a relative abundance of 82.79%, followed by *Basidiomycota* (6.06%) and *Mortierellomycota* (2.66%; *p* < 0.05). Meanwhile, *Ascomycota* was the only dominant fungal phylum (>5%) in LH (*p* < 0.05), accounting for a much higher relative abundance (97.69%) than that in FH (*p* < 0.05). In accordance with this finding, Lin et al. (2020) and Chen et al. (2021) also found that *Ascomycota* was the absolutely predominant fungus at the phylum level in Laowo dry-cured hams and Mianning hams, respectively. Besides, the relative abundances of many fungal phyla, mainly including *Basidiomycota*, *Rozellomycota*, *Chytridiomycota*, and *Glomeromycota* were significantly lower in LH than those in FH (*p* < 0.05).

At the genus level (Figure 5B), *Meyerozyma* (55.61%) was the most abundant fungus in FH (*p* < 0.05), followed by *Aspergillus* (11.67%; *p* < 0.05). While *Meyerozyma* (64.79%), *Yamadazyma* (17.76%), and *Aspergillus* (12.82%) were the dominant fungal genera (>2%) in LH (*p* < 0.05). Consistent with these findings, Lin et al. (2020) also found that *Yamadazyma*, *Meyerozyma*, and *Aspergillus* was the most abundant fungal genus in Laowo dry-cured hams ripened for 1–3 years, respectively. On the other hand, among the core fungal genera with a relative abundance >1%, the richness of *Fusarium*, *Debaryomyces*, *Marasmius*, and *Apiotrichum* were relatively higher in FH (*p* < 0.05), and *Yamadazyma* was the only more abundant fungus in LH (*p* < 0.05).

At the species level (Figure 5C), *Meyerozyma sp.*, *Aspergillus proliferans*, and *Yamadazyma triangularis* were all among the core fungi (>1%) of both FH and LH (*p* < 0.05). Besides, *Debaryomyces sp.* and *Apiotrichum sp.* were also the dominant fungi in FH (*p* < 0.05). Moreover, among the core fungal species with a relative abundance >0.5%, FH showed more abundant *Marasmius siccus*, *Debaryomyces sp.*, *Apiotrichum sp.*, *Archaeorhizomyces sp.*, and *Fusarium solani* (*p* < 0.05), whereas LH presented a higher relative abundance in *Yamadazyma triangularis* (*p* < 0.05).

### 3.5. Correlations and connections between differential core microorganisms and discriminative volatile flavor compounds

**Figure 6:**
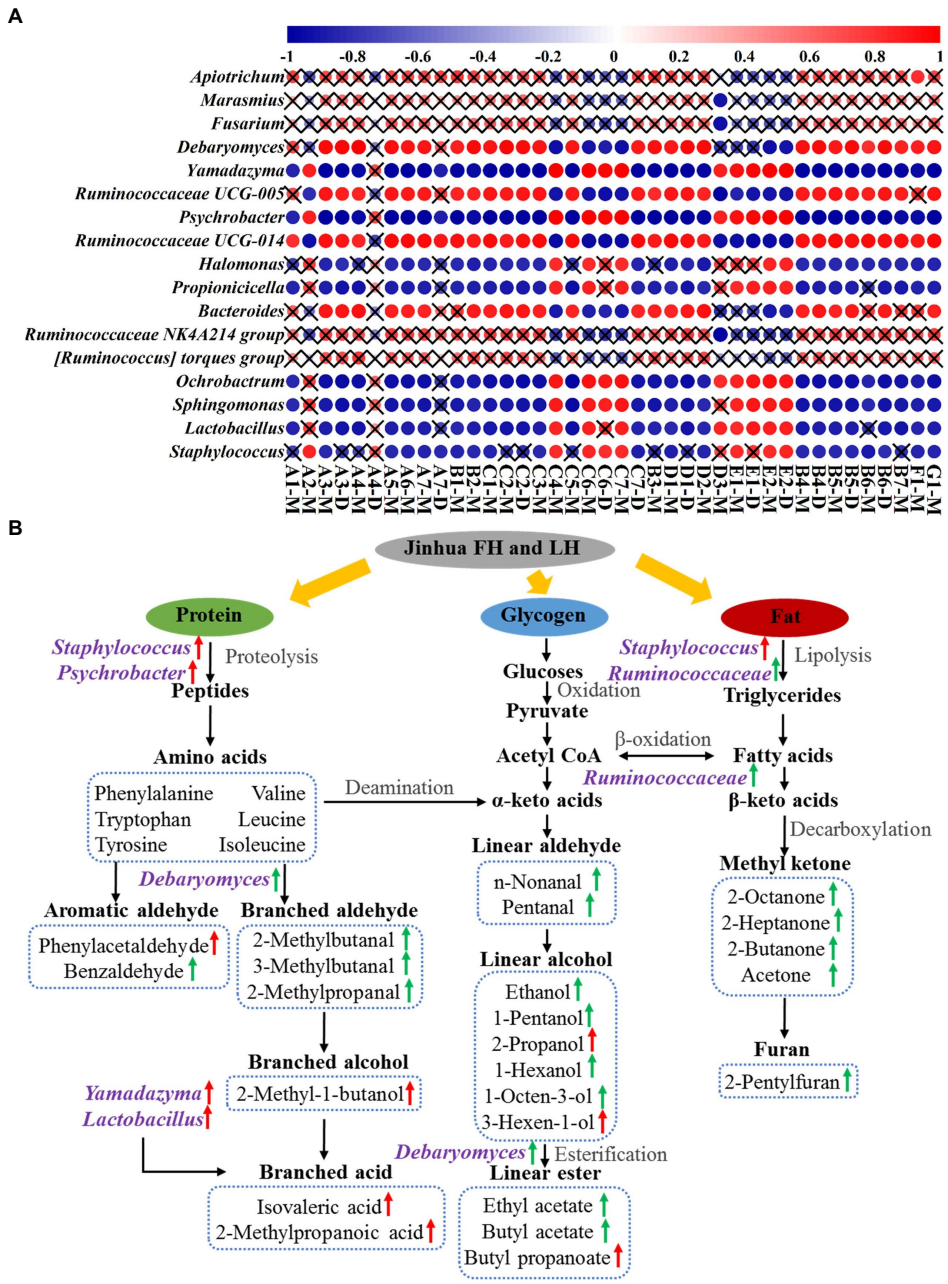
Pearson’s correlations **(A)** and potential pathways **(B)** between differential core microorganisms and distinct volatile flavor compounds. The color gradation and circle size in panel **A** represent the correlation coefficient (r value), while the pattern without a “×” symbol indicates a significant correlation (*p* < 0.05). The green and red arrows in panel **B** indicate an up-regulation in FH and LH, respectively. A1, n-nonanal; A2, phenylacetaldehyde; A3, benzaldehyde; A4, pentanal; A5, 2-methylbutanal; A6, 3-methylbutanal; A7, 2-methylpropanal; B1, 3-octanone; B2, 6-methyl-5-hepten-2-one; B3, 2-octanone; B4, 2-heptanone; B5, 3-hydroxy-2-butanone; B6, 2-butanone; B7, acetone; C1, 1-octen-3-ol; C2, 1-pentanol; C3, ethanol; C4, 2-propanol; C5, 1-hexanol; C6, 3-hexen-1-ol; C7, 3-methyl-1-butanol; D1, ethyl acetate; D2, butyl acetate; D3, butyl propanoate; E1, isovaleric acid; E2, 2-methylpropanoic acid; F1, 2-pentylfuran; G1, dimethyl disulfide; M, monomer; D, dimer.

Figure 6A shows the Pearson’s correlations between 17 differential core microbial genera (*p* < 0.05 and relative abundance >1%; 12 bacterial genera, 2 yeast genera, and 3 mold genera) and 29 discriminative volatile flavor compounds (*p* < 0.05) in Jinhua FH and LH. Surprisingly, 12 of the 17 differential core microorganisms, including 10 bacterial genera and 2 yeast genera, showed significant correlations with the distinct volatiles (*p* < 0.05 and |r| > 0.82). Among these microbial genera, *Staphylococcus*, *Lactobacillus*, *Yamadazyma*, *Ochrobactrum*, and *Sphingomonas* were one type of microbes mainly positively correlating with acids, branched alcohols, and a few linear-chain alcohols (2-propanol and 3-hexen-1-ol) in the two hams (*p* < 0.05 and r > 0.82). *Staphylococcus* has been considered the most important factor in the sensory characteristics development of dry-cured hams, owning to its strong abilities of nitrate reductase, catalase, protease, and lipase (Landeta et al., 2013; Xiao et al., 2020). *Lactobacillus* could extend the shelf life of hams by inhibiting the growth of spoilage bacteria *via* its product lactic acid, which could be further utilized by other microorganisms for SCFA synthesis (Figure 6A; Table 1; Xiao et al., 2020; Hu et al., 2022). *Yamadazyma* has been reported positively related to the organic acid level and negatively associated with the amino acid content in hams (Lin et al., 2020; Mu et al., 2020), suggesting its potential capability of producing SCFAs from amino acids (Figure 6A). Furthermore, *Lactobacillus* and *Yamadazyma* were both core microorganisms with higher relative abundances in LH (*p* < 0.05; Figures 4B, 5B), which could be an important contributor to the relatively higher acid volatile level (Figure 1; Table 2) and lower pH (Table 1) of LH (*p* < 0.05), owning to their potential association with SCFA generation.

On the other hand, *Ruminococcaceae* UCG-005, *Psychrobacter*, *Ruminococcaceae* UCG-014, and *Debaryomyces* were another type of microorganisms that positively correlated with aldehydes, ketones, most linear-chain alcohols, esters, and the other two compounds (2-pentylfuran and dimethyl disulfide) in the two hams (*p* < 0.05 and r > 0.82). *Psychrobacter* is a common bacterium in low-temperature fermented foods with substantial proteolysis ability (Broekaert et al., 2013). Zhang et al. (2021) has reported that *Debaryomyces* could produce branched aldehydes, such as 2-methylbutanal and 3-methylbutanal, by utilizing amino acids. Furthermore, Flores et al. (2004) reported that *Debaryomyces* could promote the generation of ethyl esters in dry-fermented meats. The intensities of 2-methylbutanal, 3-methylbutanal, and ethyl acetate were all positively correlated with the richness of *Debaryomyces* (Figure 6A), which were consistent with their findings. Moreover, the role of *Ruminococcaceae* family was rarely reported in fermented meat products, but it is known with capacities of fat degradation and fatty acid β-oxidation (Baars et al., 2018), which might contribute to the high-level volatiles originated from fats in FH, such as methyl ketones, furans, and some liner-chain aldehydes/alcohols (Figure 6A).

According to the present data and associated previous reports (Chen et al., 2017; Shi et al., 2019; Mu et al., 2020; Li et al., 2022), the potential metabolic network of discriminative volatiles’ formation under the involvement of core microorganisms in Jinhua FH and LH is further illustrated in Figure 6B. Briefly, the absence of fats significantly affected the microbial community structure and the flavor formation process in LH. By lipases from *Staphylococcus* and *Ruminococcaceae* in FH, fats can be degraded into fatty acids, which further generated methyl ketones and furans through decarboxylation and other reactions. Besides, the unsaturated fatty acids can be broken down through the β-oxidation by *Ruminococcaceae* to produce linear-chain aldehydes, which can be reduced to linear-chain alcohols and further esterified under the promotion of *Debaryomyces*. Whereas in LH, the protein metabolism was relatively enhanced due to the lack of fats and the action of characteristic core microbes. Under the proteolysis of *Staphylococcus* and *Psychrobacter*, more proteins were degraded into amino acids, which were then subjected to Strecker degradation and deamination for further generation of some aromatic aldehydes or linear-chained alcohols. Besides, the branched acids could be generated by *Yamadazyma* and some other microbes through utilizing amino acids or *Lactobacillus*-produced lactic acids. In summary, this metabolic network confirmed that the discriminative flavor compounds of FH were mainly β-oxidation and degradation products of fatty acids, whereas those of LH were mostly derived from the Strecker reaction or microbial metabolism of amino acids.

## 4. Conclusion

In conclusion, the lack of fats obviously influenced the microbial composition and flavor formation of LH, which further affected some physiochemical parameters. FH and LH did not show significant differences in redness, water activity, chemical composition, and nitrite residue, but FH had higher pH and a slightly lighter and yellower color. Besides, a total of 33 volatile flavor compounds were identified. FH and LH exhibited significant differences in 29 identified volatiles, among which LH showed higher total abundance of alcohols and acids, whereas FH had generally richer aldehydes, ketones, esters, heterocyclic compounds, and sulfur-containing compounds. Meanwhile, FH and LH also showed no significant difference in α-diversity of bacterial community, but LH had a both lower richness and diversity of fungal community than FH. The dominant microorganisms (>2%) were *Ruminococcaceae* UCG-005, *Staphylococcus*, *Ruminococcaceae* UCG-014, *Meyerozyma*, and *Aspergillus* in FH at the genus level, whereas *Staphylococcus*, *Psychrobacter*, *Halomonas*, *Propionicicella*, *Ruminococcaceae* UCG-005, *Meyerozyma*, *Yamadazyma*, and *Aspergillus* for LH. The analysis of Pearson’s correlation and metabolic network confirmed that the discriminative flavor compounds of FH were mainly β-oxidation and degradation products of fatty acids, while those of LH were mostly derived from the Strecker reaction or microbial metabolism of amino acids. Furthermore, *Staphylococcus*, *Yamadazyma*, *Ruminococcaceae*, *Debaryomyces*, *Lactobacillus*, and *Psychrobacter* might be the core microorganisms contributing to their differences in flavor characteristics and pH value. This work could help understand the potential pathway of characteristic microorganisms influencing flavor formation of fat-deficient dry-cured hams and provide theoretical supports for developing healthier fermented meat products.

## Data availability statement

The data presented in the study are deposited in the Sequence Read Archive of National Center for Biotechnology Information repository (https://www.ncbi.nlm.nih.gov/sra), accession number PRJNA917481.

## Author contributions

JZ: conceptualization, methodology, writing-original draft, writing-review and editing, visualization, and funding acquisition. KZ: data curation. HL: software. SL: validation. WX: resources. LC: funding acquisition. JX: formal analysis and resources. HT: conceptualization, resources, and funding acquisition. All authors contributed to the article and approved the submitted version.

## Funding

This research was financially supported by the Zhejiang Key Research and Development Program (Nos. 2021C04024 and 2022C02060), Zhejiang Natural Science Foundation (No. LY21C200003), International Cooperation Fund of Zhejiang Academy of Agricultural Science (No. 10411040122GJ0101F), Young Professionals Promotion Funds of Zhejiang Academy of Agricultural Sciences (No. 2020R15R08E01), and Scientific Research Fund for Advanced Scholar of Zhejiang Academy of Agricultural Science (No. 3300002172744).

## Conflict of interest

WX is employed by Jinhua Jinnian Ham Co., Ltd (Jinhua, China).

The remaining authors declare that this research was conducted in the absence of any commercial or financial relationships that could be construed as a potential conflict of interest.

## Publisher’s note

All claims expressed in this article are solely those of the authors and do not necessarily represent those of their affiliated organizations, or those of the publisher, the editors and the reviewers. Any product that may be evaluated in this article, or claim that may be made by its manufacturer, is not guaranteed or endorsed by the publisher.
